# Pathogenetic Mechanisms Underlying Spinocerebellar Ataxia Type 3 Are Altered in Primary Oligodendrocyte Culture

**DOI:** 10.3390/cells11162615

**Published:** 2022-08-22

**Authors:** Kristen H. Schuster, Alexandra F. Putka, Hayley S. McLoughlin

**Affiliations:** 1 Department of Neurology, University of Michigan, Ann Arbor, MI 48109, USA; 2 Neuroscience Graduate Program, University of Michigan, Ann Arbor, MI 48109, USA

**Keywords:** spinocerebellar ataxia type 3, Machado–Joseph disease, ataxia, polyglutamine, oligodendrocyte, myelination, oligodendrocyte precursor cells

## Abstract

Emerging evidence has implicated non-neuronal cells, particularly oligodendrocytes, in the pathophysiology of many neurodegenerative diseases, including Alzheimer’s disease, Parkinson’s disease, amyotrophic lateral sclerosis, Huntington’s disease and Spinocerebellar ataxia type 3 (SCA3). We recently demonstrated that cell-autonomous dysfunction of oligodendrocyte maturation is one of the of the earliest and most robust changes in vulnerable regions of the SCA3 mouse brain. However, the cell- and disease-specific mechanisms that underlie oligodendrocyte dysfunction remain poorly understood and are difficult to isolate in vivo. In this study, we used primary oligodendrocyte cultures to determine how known pathogenic SCA3 mechanisms affect this cell type. We isolated oligodendrocyte progenitor cells from 5- to 7-day-old mice that overexpress human mutant ATXN3 or lack mouse ATXN3 and differentiated them for up to 5 days in vitro. Utilizing immunocytochemistry, we characterized the contributions of ATXN3 toxic gain-of-function and loss-of-function in oligodendrocyte maturation, protein quality pathways, DNA damage signaling, and methylation status. We illustrate the utility of primary oligodendrocyte culture for elucidating cell-specific pathway dysregulation relevant to SCA3. Given recent work demonstrating disease-associated oligodendrocyte signatures in other neurodegenerative diseases, this novel model has broad applicability in revealing mechanistic insights of oligodendrocyte contribution to pathogenesis.

## 1. Introduction

Spinocerebellar ataxia type 3 (SCA3), also called Machado–Joseph Disease, belongs to the family of neurogenerative diseases caused by autosomal dominant CAG trinucleotide expansions. In SCA3, the CAG repeat occurs in exon 10 of the *ATXN3* gene, translating to an expanded polyglutamine (polyQ) repeat in the ATXN3 protein [1,2,3]. While the number of CAG repeats in healthy individuals ranges between 12 and 44, affected individuals have at least one *ATXN3* allele with 56–87 CAG repeats [4,5,6,7]. SCA3 patients experience progressive motor impairments due to cell loss in vulnerable brain regions including the spinal cord, cerebellum, brainstem, midbrain, striatum, and thalamus [8,9,10].

Of interest in the underlying mechanism of disease are changes in the localization and function of ATXN3. ATXN3 is ubiquitously expressed throughout all cell types in the central nervous system (CNS) and was first described to function as a deubiquitinase (DUB) with roles in protein quality control pathways [10,11,12,13]. Under basal conditions, ATXN3 is localized to the cytoplasm, but cellular stress triggers rapid nuclear translocation [14]. A hallmark of SCA3 neuropathology is the nuclear accumulation of ATXN3 [2,14,15], first established in the neuronal nuclei of post-mortem patient pontine tissue [14]. Few studies have assessed the effects of ATXN3 nuclear accumulation in other CNS cell types. More recent noninvasive imaging in patients documented the loss of white matter early in SCA3 disease progression [16,17,18,19,20]. Therefore, as the myelinating white matter cells of the brain, oligodendrocytes may have an important role in early disease pathogenesis. Investigations of ATXN3 localization and function in this cell type specifically have yet to be tested.

Our lab recently demonstrated that the dysregulation of oligodendrocyte maturation is among the earliest and most robust changes in SCA3 mice [21]. Using RNA-sequencing, we identified a disease-associated weighted gene co-expression network analysis module in YACQ84 (Q84) mice that was enriched for oligodendrocyte genes. This disease module implicated the impairment of oligodendrocyte maturation that we validated through biochemical, histological, and ultrastructural analysis in Q84 mice. We further demonstrated that mutant ATXN3 acts via a toxic gain-of-function mechanism, as we observed no changes in oligodendrocyte maturation markers by biochemical and histological analysis in *Atxn3* knockout (*Atxn3*-KO) mice. Culminating this work, we found the deficit in oligodendrocyte maturation to be cell-autonomous, with primary oligodendrocyte culture from Q84 mice recapitulating the maturation impairments we see in vivo. These results reveal the oligodendrocyte signature to be an early feature of SCA3 pathogenesis and highlight the importance of investigating non-neuronal contributions to disease.

Oligodendrocyte impairments are not unique to the pathophysiology of SCA3. Research in amyotrophic lateral sclerosis (ALS), Alzheimer’s disease (AD) and Huntington’s disease (HD) have all reported significant pathogenic oligodendrocyte signatures in disease progression [22,23,24,25,26,27]. For example, Kang et al. (2013) demonstrated, in an ALS model, the extensive degeneration of oligodendrocytes in the spinal cord of presymptomatic *SOD1* mice, along with the increased proliferation of NG2+ cells. Similar to our results in SCA3, this study also found an oligodendrocyte maturation failure in *SOD1* mice [28]. A recent paper in *Nature Neuroscience* highlighted a disease-associated oligodendrocyte signature in multiple AD mouse models [27]. In addition, there is a large body of evidence supporting the role of oligodendrocytes in the pathogenesis of HD, another CAG repeat expansion disorder [26,29,30,31]. One study showed that abnormalities in myelin preceded behavioral deficits and neuron death in a mouse model of HD [26]. By conditionally reducing mutant HTT in oligodendrocyte lineage cells in HD mice, the authors were able to rescue several behavioral deficits [26]. Together, these studies indicate that disease-associated oligodendrocyte signatures are a common feature of neurodegeneration that must be further investigated.

Our previous study demonstrated the cell-autonomous defect in oligodendrocyte maturation using primary oligodendrocyte cell culture from Q84 SCA3 mice [21]. What remains unknown is the potential for this cell culture model to inform the molecular pathways underlying this SCA3 phenotype. We hypothesize the maturation impairments observed in SCA3 oligodendrocytes occur from common pathomechanisms of SCA3 disease. To test this hypothesis, we characterized known mechanisms of SCA3 in primary oligodendrocytes from Q84 and *Atxn3*-KO mice relative to their respective wild-type (WT) littermates. Few studies have investigated the effects of SCA3 disease on protein quality control in a cell-specific manner. Therefore, we characterize key protein changes of pathways known to be disrupted in SCA3 within the general oligodendrocyte population, including the ubiquitin–proteasome system and autophagy. Because DNA damage and the methylation of histone 3 could affect the typical oligodendrocyte differentiation process, we distinguish between immature and mature cells to decipher potential dysregulated mechanisms influencing maturation state. We demonstrate both gain- and loss-of-function alterations in these pathways. Our findings illuminate the cell-autonomous characteristics of oligodendrocytes in SCA3 and emphasize the utility of primary oligodendrocyte for mechanistic and translational research.

## 2. Materials and Methods

### 2.1. Mice

All animal procedures were approved by the University of Michigan Institutional Animal Care and Use Committee (PRO00010182; date of approval: 3/15/2021) and conducted in accordance with the United States Public Health Service’s Policy on Humane Care and Use of Laboratory Animals. Genotyping was performed using tail biopsy DNA isolated prior to weaning and confirmed postmortem, as previously described [32]. Genotyping for the YACQ84 line was previously described [33]. Genotyping for the *Atxn3*-KO line [34] was completed using *Atxn3*-KO Forward 5′-GAGGGAAGTCGTCATAAGAGT-3′, *Atxn3*-KO Reverse 5′-TGGGCTACAAGAAATCCTGTC-3′, and *Atxn3*-KO LTRa 5′-AAATGGCGTTACTTAAGCTAG-3′ primers.

### 2.2. OPC Isolation and Culture

As previously described [21], magnetic cell sorting (MACS, Miltenyi Biotec, Bergisch Gladbach, Germany) was used to isolate oligodendrocyte precursor cells (OPCs) from the whole brains of 5–7-day old YACQ84 or *Atxn3*-KO mice. Briefly, whole brains were dissected and homogenized into a single cell suspension. Magnetic PDGFRα microbeads (Miltenyi Biotec, Bergisch Gladbach, Germany, #130-101-502) were used to label OPCs, which were then isolated using LS columns (Miltenyi Biotec, Bergisch Gladbach, Germany, #130-042-401). Cells were plated in OPC media (+FGF2, +PDGFAA) on 8-well slides at 5 × 10^3^ per mm^2^ or 4-well slides at 2.5 × 10^3^ per mm^2^. The next day, cells were subjected to either 4% PFA fixation for 20 min at room temperature (DIV0) or switched to differentiation media (OPC media, -FGF2, -PDGFAA, +T3) for fixation 3 days (DIV3) or 5 days (DIV5) later. Differentiation media were changed every other day.

### 2.3. Immunocytochemistry

Oligodendrocytes were stained as previously described [21]. Briefly, cells were washed with PBS, blocked with normal goat serum, permeabilized with Triton X-100, then incubated in primary antibody overnight at 4 °C. The primary antibodies used are listed in Table 1. The following day, cells were incubated with corresponding AlexaFluor488, 568, or 647 secondary antibodies (1:1000, Invitrogen, Waltham, MA, USA) for 1 h at room temperature and co-stained with DAPI. Slides were cover-slipped with Prolong Gold Antifade Reagent (Invitrogen) and imaged using a Nikon-A1 Standard Sensitivity confocal microscope with NIS-Elements software. Four-to-eight images were randomly taken in each well, with 1–2 wells per mouse, and 2–5 mice per genotype.

### 2.4. Image Analysis

Images were analyzed using CellProfiler software [35]. Cell counts were obtained by assessing the overlay of the protein marker of interest and DAPI-stained nuclei in each image, taken as a percentage of total DAPI-stained nuclei, and normalized to the average of WT/WT counts. The protein expression was measured using the intensity of fluorescence. All images were co-stained with DAPI, therefore nuclear intensity was analyzed by measuring the fluorescence in DAPI outlined nuclei. Whole-cell intensity was analyzed by delineating each cell with an outline and quantifying the fluorescence within. Intensity was averaged per cell for each image and images were normalized to WT/WT average.

### 2.5. Statistics

All statistical analyses were performed using Prism (GraphPad Software 9.0, La Jolla, CA, USA) using either a Student’s *t*-test or a one-way ANOVA with a post hoc Tukey’s multiple comparisons test. Data are reported as the mean ± standard error of the mean (SEM) and all statistical tests are set to a *p* < 0.05 level of significance.

## 3. Results

### 3.1. Cell Autonomous SCA3 Oligodendrocyte Maturation Is Impaired via a Toxic Gain-of-Function Mechanism

Among the neurodegenerative diseases, the dominantly inherited polyglutamine repeat expansion disorders have pointed toward a significant role of oligodendrocytes in disease pathogenesis [10,21,22,23,24,25,26,27]. Our lab has established the importance of oligodendrocytes early in SCA3 pathogenesis [21] and highlighted the importance of therapeutically targeting this cell type [33,36]. However, the intrinsic mechanisms that underlie this cell-autonomous defect in SCA3 are not well understood. Here, we characterize the gain of toxic ATXN3 function compared to the loss of ATXN3 function in oligodendrocytes by culturing primary oligodendrocytes from Q84 mice and *Atxn3*-KO mice.

**Figure 1:**
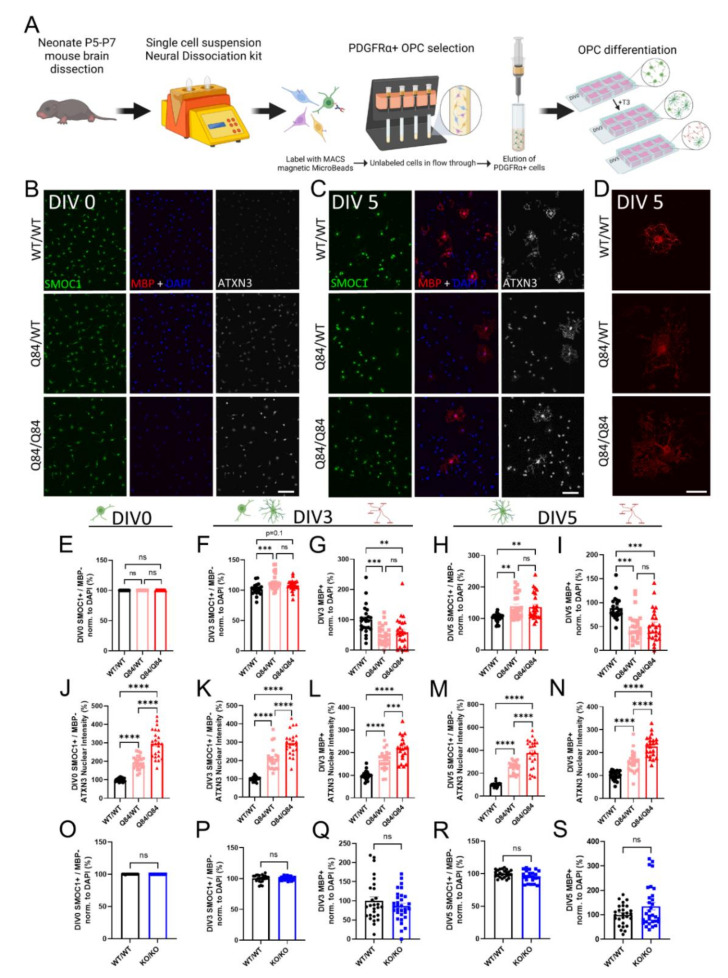
ATXN3 gain of toxic function, but not loss of function, leads to cell-autonomous oligodendrocyte maturation impairments. (**A**) Schematic of primary OPC isolation and culture. (**B**,**C**) Representative immunofluorescent images of SMOC1 (green), MBP (red), ATXN3 (white), and DAPI (blue) expression in cultured WT/WT, Q84/WT, and Q84/Q84 oligodendrocytes prior to differentiation (DIV0) (**B**) and after 5 days of differentiation (+T3, DIV5) (**C**). Scale bar, 100 µm. (**D**) Representative high magnification images of MBP staining of cultured WT/WT, Q84/WT, and Q84/Q84 oligodendrocytes depict irregular branching in Q84 cells. Scale bar: 25 µm. (**E**,**F**,**G**) Cell counts of immature oligodendrocytes (SMOC1+/MBP−) at DIV0 (**E**), DIV3 (**F**), and DIV5 (**H**). (**G**,**I**) Cell counts of mature oligodendrocytes (MBP+) at DIV3 (**G**) and DIV5 (**I**). No differences in cell counts were found at DIV0, however, at DIV3 and DIV5, immature oligodendrocytes were increased and mature oligodendrocytes were significantly decreased in diseased mice. (**J**) Quantification of average ATXN3 nuclear intensity in Q84 OPCs at DIV0. (**K**–**N**) Quantification of average ATXN3 intensity in Q84 immature and mature oligodendrocyte nuclei at DIV3 (**K**,**L**) and DIV5 (**M**,**N**). Oligodendrocytes from diseased mice, regardless of maturation state, show a dose-dependent increase in nuclear ATXN3 accumulation. (**O**–**S**) Cell counts of OPCs cultured from *Atxn3*-KO mice at DIV0 (**O**), of immature and mature oligodendrocytes at DIV3 (**P**,**Q**) and at DIV5 (**R**,**S**). Cell counts of immature and mature oligodendrocytes are not different in *Atxn3*-KO mice compared to WT at all assessed timepoints. Cell counts taken as a percentage of the total DAPI-stained nuclei per field and normalized to WT/WT; Intensity quantifications normalized to averaged WT/WT images (n = 4–8 images per mouse, n = 4–5 mice per genotype). Data presented as mean ± SEM. One-way ANOVA with Tukey’s multiple comparisons test or Student’s *t*-test were performed: ns = not significant; ** *p* < 0.01; *** *p* < 0.001; **** *p* < 0.0001.

Oligodendrocytes are thought to develop through three stages—OPCs, immature (pre-myelinating) oligodendrocytes, and mature myelinating oligodendrocytes [37,38]. In order to isolate sufficient OPCs for experimental differentiation analysis, oligodendrocytes need to be captured before differentiation begins. The highest rate of myelination in mice occurs between post-natal days 7 and 21 [39], therefore we isolated OPCs from mice between 5- and 7-days-old. Whole mouse brains were dissociated into a single cell suspension and labeled with a magnetically conjugated antibody against the OPC-specific receptor, PDGFRα (Figure 1A). Labeled cells were captured in a magnetic column, eluted, and cultured in OPC proliferation media (+FGF2, +PDGFAA) for one day on chamber slides. Cells were either fixed prior to differentiation (DIV0) or switched to differentiation media (OPC media, −FGF2, −PDGFAA, +T3) for collection 3 or 5 days later (DIV3, DIV5) (Figure 1A).

Using these cultures, we first verified the previous findings of a cell-autonomous maturation defect in SCA3 oligodendrocytes at DIV0 and DIV3 [21], and determined whether this impairment extends to a later timepoint (DIV5) that allowed more time for cells to mature. We used SMOC1 as a marker of the OPC state [12,21,36] and confirmed no differences in the maturation state after plating SCA3 OPCs (Figure 1B,E). Using a marker for the mature myelinating state (MBP), we continued to observe a decrease in mature Q84 oligodendrocytes at DIV3 with an increasing trend in immature oligodendrocytes (SMOC1+; MBP− cells) (Figure 1F,G). These trends persisted at DIV5 (Figure 1C), with a significant decrease in mature Q84 oligodendrocytes and a significant increase in immature oligodendrocytes relative to WT cultures (Figure 1H,I). Of note, SCA3 oligodendrocytes that are able to reach a mature state at DIV5 were consistently observed to be cytoarchitecturally abnormal (Figure 1D). Mature WT oligodendrocytes at this timepoint were organized in their branching, with the expected pruning of unnecessary branches and end feet for myelination [40,41,42], whereas diseased mature oligodendrocytes display unorganized and excessive branching (Figure 1D). Regardless of days in vitro or maturation state, Q84 oligodendrocytes showed a disease dose-dependent increase in nuclear ATXN3 accumulation (Figure 1J–N), a hallmark of SCA3 disease. Importantly, when we isolated OPCs from *Atxn3*-KO mice, we found no differences in the immature or mature oligodendrocyte cell counts at all timepoints assessed (Figure 1O–S and Figure S1). This implies that the impairment impeding oligodendrocyte maturation in SCA3 is not due to ATXN3 loss-of-function, but rather it is likely caused by a gain of toxic function of mutant ATXN3. We therefore utilized oligodendrocytes from *Atxn3*-KO mice throughout the remainder of this study to distinguish between loss-of-function mechanisms and those that contribute to the maturation defect in SCA3 oligodendrocytes.

A lack of mature oligodendrocytes in disease could be attributed to various biological processes, two of the most obvious being cell proliferation and death. SCA3 OPCs may be proliferating or mature SCA3 oligodendrocytes may be dying at higher rates than WT oligodendrocytes. However, after quantifying the number of OPCs at DIV0 and DIV5 positive for Ki-67, a cell proliferation marker, we found no differences in proliferating cells (data not shown). We also saw no changes in total cell counts (DAPI+ cells) between genotypes at each timepoint, suggesting there is no overt cell death (data not shown). Taken together, these data imply that the increase in immature oligodendrocytes and decrease in mature oligodendrocytes is not caused by the disproportionate proliferation or death of oligodendrocyte lineage cells. Oligodendrocytes are complex cells that can be affected by a variety of both intrinsic and extrinsic factors [40,43], yet SCA3 oligodendrocytes are not overtly affected by the influences of cell death or proliferation. We therefore turned inward to evaluate the role of ATXN3 in canonical pathways relevant to SCA3 disease and oligodendrocyte maturation.

### 3.2. Protein Ubiquitination Is Dysregulated in Both SCA3 and Atxn3-KO Oligodendrocytes

**Figure 2:**
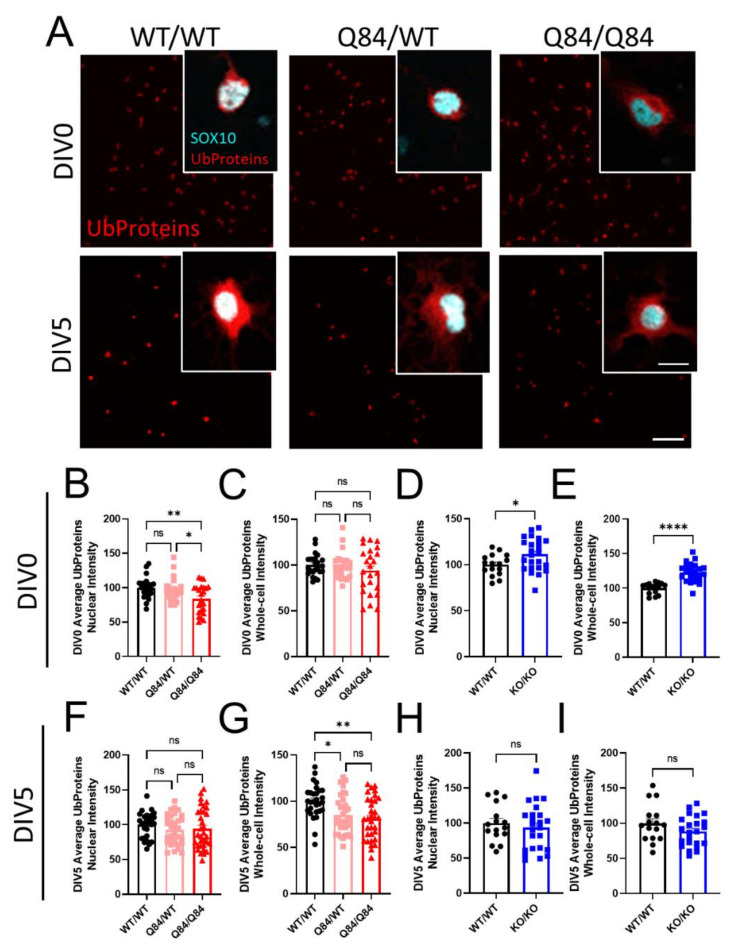
Fewer ubiquitinated proteins are present in SCA3 oligodendrocytes. (**A**) Representative immunofluorescent images stained for ubiquitinated proteins (red; Ub proteins) and pan-oligodendrocyte marker SOX10 (cyan) in WT/WT, Q84/WT, and Q84/Q84 oligodendrocytes at DIV0 and DIV5. Scale bar: 100 µm; inset scale bar: 12.5 µm (**B**–**E**) Quantification of DIV0 nuclear and whole-cell ubiquitinated protein intensity in WT and Q84 oligodendrocytes (**B**,**C**) and in WT and *Atxn3*-KO oligodendrocytes (**D**,**E**). Nuclear intensity of ubiquitinated proteins at DIV0 is decreased in Q84/Q84 oligodendrocytes, with no changes in whole-cell intensity. Both the nuclear and whole-cell intensities of ubiquitinated proteins are increased at DIV0 in *Atxn3*-KO oligodendrocytes. (**F–I**) Quantification of DIV5 nuclear and whole-cell ubiquitinated protein intensity in WT and Q84 oligodendrocytes (**F**,**G**) and in WT and *Atxn3*-KO oligodendrocytes (**H**,**I**). At DIV5, the whole-cell intensity of ubiquitinated proteins is decreased in Q84 mice relative to WT while nuclear intensity is unchanged. *Atxn3*-KO oligodendrocytes at DIV5 show no differences in ubiquitinated protein levels compared to WT cultures. Intensity quantifications normalized to averaged WT/WT images (n = 4–8 images per mouse, n = 2–5 mice per genotype). Data presented as mean ± SEM. One-way ANOVA with Tukey’s multiple comparisons test or Student’s *t*-test were performed: ns = not significant; * *p* < 0.05; ** *p* < 0.01; **** *p* < 0.0001.

As endogenous ATXN3 functions as a DUB, we began our investigations of characteristic SCA3 dysfunction by looking at the ubiquitin-dependent proteosome pathway. To understand the role of ATXN3 as a DUB in oligodendrocytes specifically, we stained SCA3 and *Atxn3*-KO oligodendrocytes at DIV0 and DIV5 for poly-ubiquitinated proteins (Figure 2 and Figure S2). We further utilized the 2D cell culture system to analyze the sub-cellular localization of ubiquitinated proteins by distinguishing between nuclear and whole-cell intensity. Unexpectedly, we found that in SCA3 OPCs, levels of ubiquitinated proteins are significantly decreased in the nucleus at DIV0 relative to WT, with no changes in whole-cell analysis (Figure 2A–C). At DIV5, the localization of ubiquitinated protein switches in that nuclear intensity is not changed, while the whole-cell intensity of ubiquitinated substrates is decreased in Q84 oligodendrocytes compared to WT (Figure 2A,F,G). If ATXN3 functions as a DUB in oligodendrocytes, but in disease is sequestered in the nucleus and unable to perform that function, we would expect levels of ubiquitinated proteins to increase as they do in vivo [33]. However, we see the opposite. Interestingly, *Atxn3*-KO oligodendrocytes display an increased intensity of ubiquitinated proteins at DIV0 but return to WT levels at DIV5 (Figure 2D,E,H,I and Figure S2). This suggests there could be a compensatory mechanism for deubiquitination. It is also possible that the primary function of ATXN3 in oligodendrocytes is not a DUB. The role of ATXN3 in oligodendrocytes specifically has been completely unexplored, and these results show that it may not be as simple as previously thought.

### 3.3. Autophagy Is Not Affected in SCA3 Oligodendrocytes

**Figure 3:**
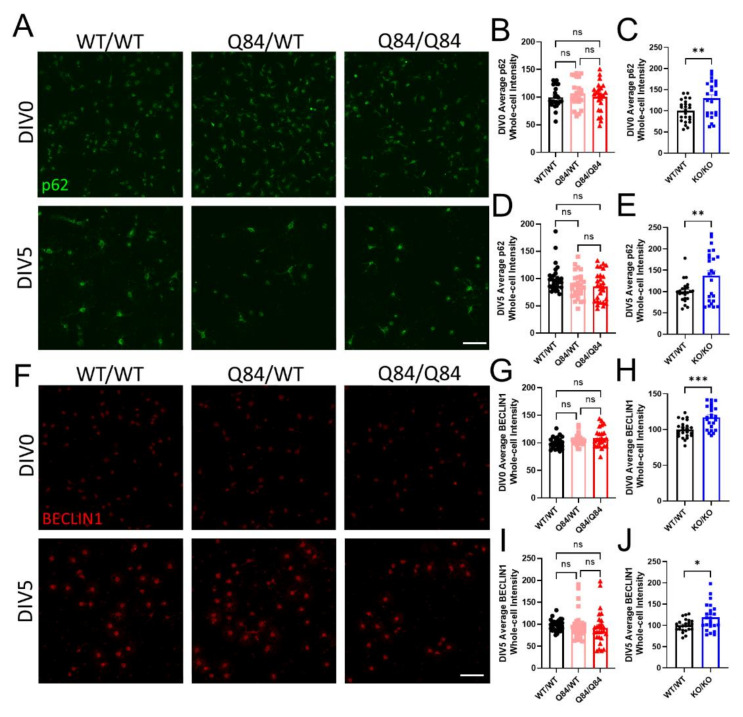
SCA3 oligodendrocytes have no changes in autophagy while the loss of ATXN3 function leads to autophagic dysregulation. (**A**) Representative immunofluorescent images of p62 in WT/WT, Q84/WT, and Q84/Q84 oligodendrocytes at DIV0 and DIV5. Scale bar: 100 µm. (**B**–**E**) Quantification of whole-cell p62 expression at DIV0 and DIV5. Whole-cell p62 intensity in Q84 oligodendrocytes remains similar to WT levels at both DIV0 (**B**) and DIV5 (**D**), but is increased in *Atxn3*-KO oligodendrocytes relative to WT at both timepoints (**C**,**E**). (**F**) Representative immunofluorescent images of BECLIN1 in WT/WT, Q84/WT, and Q84/Q84 oligodendrocytes at DIV0 and DIV5. Scale bar: 100 µm. (**G**–**J**) Quantification of whole-cell BECLIN1 expression at DIV0 and DIV5. Whole-cell BECLIN1 is unchanged in Q84 oligodendrocytes at both DIV0 (**G**) and DIV5 (**I**) and is increased in *Atxn3*-KO oligodendrocytes relative to WT at both timepoints (**H**,**J**). Intensity quantifications normalized to averaged WT/WT images (n = 4–8 images per mouse, n = 3–5 mice per genotype). Data presented as mean ± SEM. One-way ANOVA with Tukey’s multiple comparisons test or Student’s *t*-test were performed: ns = not significant; * *p* < 0.05; ** *p* < 0.01; *** *p* < 0.001.

The ubiquitin–proteosome system is one of the two protein quality control pathways in cells. The other, autophagy, utilizes the lysosome for protein degradation. Both pathways are often affected in SCA3, as protein misfolding is a common contributor to disease pathogenesis [10]. To determine the overall changes in protein quality control in Q84 and *Atxn3*-KO oligodendrocytes, we quantified whole-cell intensity levels of p62 (Figure 3A), a shuttling protein involved in both pathways [44].

We found that at DIV0 and DIV5, p62 expression levels were unchanged in Q84 oligodendrocytes (Figure 3A,B,D). Interestingly, in *Atxn3*-KO oligodendrocytes, there was an increase in p62 levels at both timepoints (Figure 3C,E and Figure S3A). In combination, these results suggest that the gain in toxic function of mutant ATXN3 in disease has no effect on overall protein quality control in oligodendrocytes, but the loss of ATXN3 function in *Atxn3*-KO oligodendrocytes leads to the upregulation of one or both of these pathways.

To measure the impact of ATXN3 on autophagy alone in oligodendrocytes, we quantified whole-cell BECLIN1 intensity at DIV0 and DIV5. At both timepoints assessed, we again found no variance in BECLIN1 protein expression in Q84 oligodendrocytes compared to WT, suggesting that autophagy is not dysregulated in SCA3 oligodendrocytes (Figure 3F,G,I). In contrast, *Atxn3*-KO oligodendrocytes had increased whole-cell expression of BECLIN1, validating the upregulation of autophagy in the absence of ATXN3 (Figure 3H,J and Figure S3B). It is important to note that previous literature demonstrating direct interactions between ATXN3 and BECLIN1 have shown that in ATXN3-depleted systems, autophagy is often downregulated [45,46,47]. To date, there are no other published reports assessing either pathway in isolated SCA3 and *Atxn3*-KO oligodendrocytes. Although unexpected, our data highlight the significance of examining individual pathways in a cell-specific manner and across maturation state in future studies.

The lack of SCA3-like phenotypes at the molecular level in isolated oligodendrocyte populations suggests that these pathways are not affected by ATXN3 gain of toxic functions. Furthermore, ATXN3 loss-of-function leads to the dysregulation of protein quality control. Therefore, these pathways likely do not contribute to the cell-autonomous SCA3 oligodendrocyte maturation impairment.

### 3.4. DNA Damage Does Not Play a Role in SCA3 Oligodendrocyte Maturation Impairments

**Figure 4:**
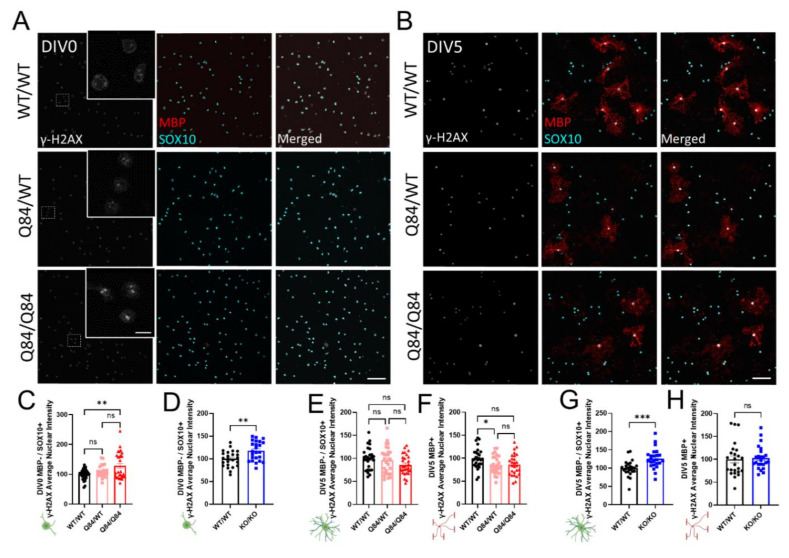
DNA damage signaling does not correlate with SCA3 oligodendrocyte maturation impairments, but may increase with cellular stress. (**A**,**B**) Representative immunofluorescent images of nuclear DNA damage marker γ-H2AX (white), mature oligodendrocyte marker MBP (red), and pan-oligodendrocyte marker SOX10 (cyan) expression in cultured WT/WT, Q84/WT, and Q84/Q84 oligodendrocytes at DIV0 (**A**) and DIV5 (**B**). Scale bar 100 µm, inset scale bar: 12.5 µm. (**C**,**D**) Quantification of nuclear γ-H2AX in DIV0 OPCs. Nuclear γ-H2AX intensity at DIV0 is increased in Q84/Q84 oligodendrocytes relative to WT (**C**) and *Atxn3*-KO oligodendrocytes relative to WT (**D**). (**E**–**H**) Quantification of nuclear γ-H2AX in immature (MBP−/SOX10+) and mature (MBP+) oligodendrocytes at DIV5. Nuclear γ-H2AX intensity is not significantly changed in immature diseased oligodendrocytes (**E**), nor in Q84/Q84 mature oligodendrocytes relative to WT. Loss of ATXN3 leads to changes in γ-H2AX nuclear expression only in immature oligodendrocytes (**G**), but not mature cells. Intensity quantifications normalized to averaged WT/WT images (n = 4–8 images per mouse, n = 3–5 mice per genotype). Data presented as mean ± SEM. One-way ANOVA with Tukey’s multiple comparisons test or Student’s *t*-test were performed: ns = not significant; * *p* < 0.05; ** *p* < 0.01; *** *p* < 0.001.

As a highly metabolic cell type, oligodendrocytes are prone to oxidative stress-induced DNA damage [48,49]. Interestingly, mutant ATXN3 has been shown to interact with essential DNA strand break repair enzymes [50,51,52], implicating DNA damage in the pathogenesis of SCA3. To evaluate the role of DNA damage in obstructing oligodendrocyte maturation in SCA3, we quantified the nuclear expression of γ-H2AX, a characteristic indicator of double-strand DNA damage [53]. To distinguish between maturation states, we co-stained with SOX10, a marker of all oligodendrocyte maturation states, and MBP, a marker of mature myelinating oligodendrocytes only (Figure 4).

At DIV0, we observed an increase in the γ-H2AX expression in SCA3 OPCs (Figure 4A,C), as well as in *Atxn3*-KO OPCs (Figure 4D and Figure S4A). By DIV5, γ-H2AX expression returned to levels similar to WT in immature and mature Q84/Q84 oligodendrocytes, though protein expression in mature Q84/WT oligodendrocytes is slightly decreased (Figure 4B,E–H). In KO cells at DIV5, γ-H2AX expression remained elevated in immature oligodendrocytes but returned to WT levels in mature oligodendrocytes (Figure 4B,G,H and Figure S4B). Because γ-H2AX expression is increased at DIV0 and returns to normal levels by DIV5 in SCA3 oligodendrocyte cultures, DNA damage signaling may be upregulated due to the cellular stress of the extraction and isolation process. The result in *Atxn3*-KO cells is consistent with literature support for ATXN3’s role in regulating DNA damage [51]. Importantly, increased nuclear γ-H2AX expression is unlikely to be associated with a failure of immature oligodendrocytes to differentiate, as KO cells matured similarly to WT (Figure 1O–S and Figure S1). In agreement, we did not see differences in γ-H2AX nuclear expression between maturation states in SCA3 oligodendrocytes relative to WT; therefore, DNA damage is unlikely to facilitate SCA3 differentiation defects.

### 3.5. Histone Methylation Is Impaired in Proliferating and Maturing SCA3 Oligodendrocytes

**Figure 5:**
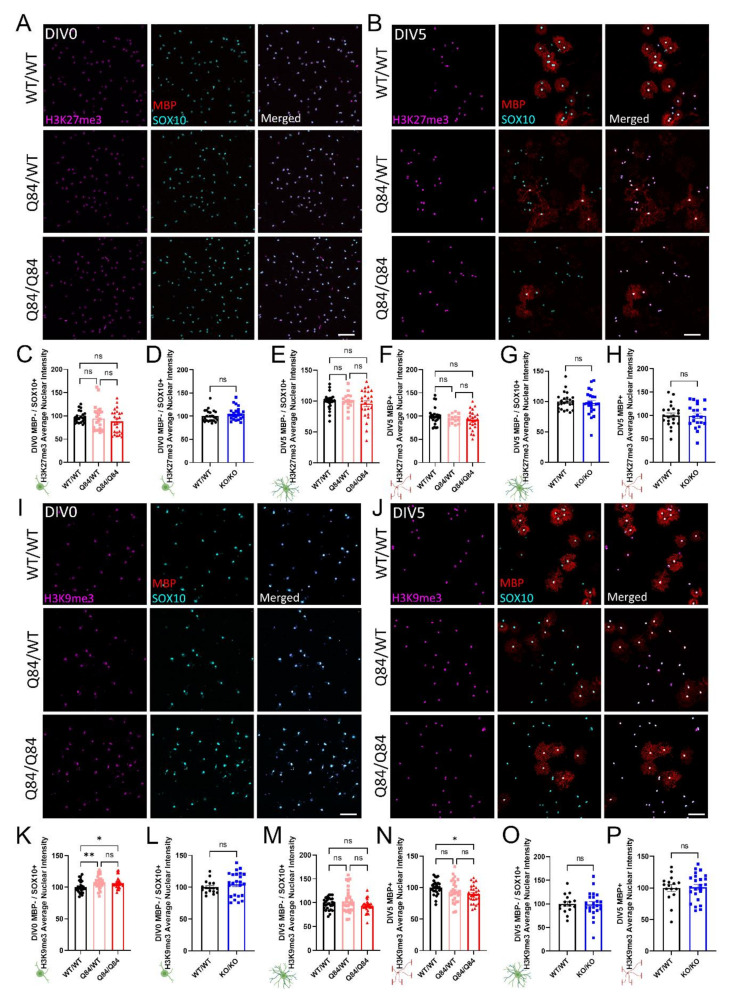
ATXN3 toxic gain-of-function in SCA3 oligodendrocytes leads to maturation-state dependent changes in the tri-methylation of H3K9, but not H3K27. (**A**,**B**) Representative immunofluorescent images of H3K27me3 (magenta), mature oligodendrocyte marker MBP (red), and pan-oligodendrocyte marker SOX10 (cyan) in WT/WT, Q84/WT, and Q84/Q84 oligodendrocytes at DIV0 (**A**) and DIV5 (**B**). Scale bar: 100 µm. (**C**,**D**) Quantification of nuclear H3K27me3 intensity of DIV0 OPCs show no changes in SCA3 oligodendrocytes (**C**) or *Atxn3*-KO oligodendrocytes (**D**). (**E**–**H**) Quantification of nuclear H3K27me3 intensity at DIV5 also shows no differences between genotypes in immature (MBP−/SOX10+) or mature (MBP+) oligodendrocytes in diseased (**E**,**F**) or *Atxn3*-KO oligodendrocytes (**G**,**H**). (**I**,**J**) Representative immunofluorescent images of H3K9me3 (magenta), MBP (red), and SOX10 (cyan) in WT/WT, Q84/WT, and Q84/Q84 oligodendrocytes at DIV0 (**I**) and DIV5 (**J**). Scale bar: 100 µm. (**K**,**P**) Quantification of nuclear H3K9me3 intensity at DIV0 and DIV5. DIV0 Q84 OPCs have increased nuclear H3K9me3 intensity (**K**). By DIV5, immature oligodendrocytes (MBP−/SOX10+) show no significant difference (**M**), but H3K9me3 nuclear intensity in mature Q84/Q84 oligodendrocytes (MBP+) has decreased relative to WT (**N**). *Atxn3*-KO oligodendrocytes show no differences of nuclear H3K9me3 intensity in DIV0 OPCs (**L**), nor in immature or mature oligodendrocytes at DIV5 (**O**,**P**). Intensity quantifications normalized to averaged WT/WT images (n = 4–8 images per mouse, n = 2–5 mice per genotype). Data presented as mean ± SEM. One-way ANOVA with Tukey’s multiple comparisons test or Student’s *t*-test were performed: ns = not significant; * *p* < 0.05; ** *p* < 0.01.

Another important regulatory pathway for oligodendrocyte differentiation is the methylation of histone 3 [54,55,56]. In particular, the trimethylation of lysine residues 9 and 27 on histone 3 (H3K9me3 and H3K27me3, respectively) has been shown to play a key part in regulating OPC differentiation into mature, myelinating oligodendrocytes by transcriptionally repressing neuronal differentiation genes [55,56]. Interestingly, H3K27me3 and H3K9me3 repression, though similar, are not redundant in their roles during OPC maturation [56], therefore it is necessary to look at the expression of both. To do this, we probed for the nuclear expression of H3K27me3 and H3K9me3 via immunocytochemistry in OPCs, as well as in immature and mature oligodendrocytes (Figure 5A,B,I,J).

When compared to WT cultures, H3K27me3 expression in both Q84 and *Atxn3*-KO cells were not different at either timepoint assessed (Figure 5A–H and Figure S5A,B). Consistent with previous oligodendrocyte maturation literature, H3K27me3 expression increased over time in WT, Q84, and *Atxn3*-KO lines as oligodendrocytes differentiated (Figure 5A,B and Figure S5A,B; data not shown).

In contrast, the expression of H3K9me3 does change in a disease-relevant, maturation state-dependent manner. At DIV0, H3K9me3 is increased in Q84 OPCs relative to WT OPCs (Figure 5I,K). The quantification of H3K9me3 expression at DIV5 reveals it is not changed in immature oligodendrocytes, but is downregulated in mature oligodendrocytes (Figure 5J,M,N). *Atxn3*-KO oligodendrocytes did not show any changes in expression of H3K9me3 at either timepoint relative to WT cells (Figure 5L,O,P and Figure S5C,D), indicating this dysregulation is specific to SCA3 disease. These results align with recent studies that demonstrated the inhibition of H3K9me3 hindered oligodendrocyte differentiation, whereas silencing of H3K27me3 had no effect [56]. Thus, the dysregulation of H3K9me3 in SCA3 oligodendrocytes may play an essential role in the impairment of oligodendrocyte maturation.

## 4. Discussion

Evidence of oligodendrocyte dysfunction has recently expanded beyond classic demyelinating diseases into neurodegenerative diseases, including ALS, Alzheimer’s disease, Huntington’s disease, and SCA3 [21,22,23,24,25,26,27,57,58,59]. The underlying mechanisms of this dysfunction, however, remain poorly understood. The primary culture of oligodendrocytes can be a powerful tool in understanding cell-specific pathways and disease mechanisms. In this study, we utilized the primary cell culture of SCA3 and *Atxn3*-KO oligodendrocytes to assess the cell-specific contributions of ATXN3 gain of toxic function and loss-of-function to pathways known to be involved in SCA3 pathogenesis and oligodendrocyte maturation.

By recapitulating our findings on cell-autonomous SCA3 oligodendrocyte maturation impairments at DIV3 and extending this measurement to DIV5, we established primary oligodendrocyte culture as a representative model of in vivo SCA3 oligodendrocyte dysfunction [21]. We further characterized this dysfunction by comparing Q84 cultures to *Atxn3*-KO and found the maturation defect to be disease-specific and due to the gain of toxic function of mutant ATXN3. Establishing this distinction allowed us to compare Q84 oligodendrocytes and *Atxn3*-KO oligodendrocytes to identify the pathways affected by mutant ATXN3 that could contribute to the maturation impairment in SCA3.

We found an intriguing lack of differences in maturation defects in our hemi and homozygous SCA3 oligodendrocyte lines. Specifically, we know this to be a dose-dependent gain of toxic function phenotype in vivo based on our previous studies [21] and gene silencing work [36]. It would then be expected that the clear dose-dependent nuclear accumulation of ATXN3 in SCA3 oligodendrocytes would correlate with maturation impairments. Our findings, however, do not match this expectation. Future assessments of mutant *ATXN3* gene silencing in oligodendrocyte cell culture will inform the level of toxic protein expression necessary to elicit the oligodendrocyte maturation impairments we report here. In addition, these studies may also tell us more about the role mutant ATXN3 toxic gain-of-function is playing in this cell population.

ATXN3 is widely recognized as a DUB; therefore, changes in the functionality of this protein would be expected to alter protein quality control pathways such as the ubiquitin–proteasome system and autophagy [33]. The results presented here are contrary to what would be expected from gain-of-function mutations of a DUB, highlighting the need to study disease protein functions in individual cell populations. Little is known about protein quality control pathways in glial cells and how they are affected in SCA3. Proteasomal and autophagic regulators need to be further explored in these isolated cell types. With regard to oligodendrocytes in particular, the state of maturation could play a role in the regulation of these pathways; i.e., OPCs or immature cells could manage protein quality differently than mature oligodendrocytes. By analyzing oligodendrocytes in culture, we are able to assess specific processes in isolated cell populations to better understand how these pathways function normally and how they become dysfunctional in disease.

The results presented here allude to the possibility of ATXN3 primarily functioning in other cellular processes. The lack of ATXN3 has been linked to disorganization of the cytoskeleton [60], and microtubule dysregulation has been demonstrated in a knock-in mouse model of SCA3 [61]. Moreover, a loss of the microtubule-associated protein Tau was demonstrated to affect oligodendrocyte differentiation and the formation of neuron-glia contact, consequently impairing myelination [41,62]. Tau pathology is a common feature in several neurodegenerative diseases [63]; therefore, studies investigating the role of tau in oligodendrocytes specifically may provide mechanistic insight toward disease-associated oligodendrocyte signatures. Consistent with the studies implicating ATXN3 in cytoskeleton organization, our cultured SCA3 mature oligodendrocytes display aberrant branching in comparison to WT cells. Furthermore, SCA3 phenotypes stemming from the inability of oligodendrocytes to mature, including excessive branching and thinner myelin sheaths [21], mirror those seen in oligodendrocytes lacking the microtubule-interacting Golgi outpost protein, TPPP [64]. Further analyses will need to quantitatively evaluate the branching and cytoskeletal regulation of these cells. Additional future mechanistic studies should also explore alternative cell-specific ATXN3 functions, particularly in the cytoarchitectural organization of oligodendrocytes.

ATXN3 has also been shown to play a role in DNA damage, a common pathological symptom of neurodegenerative diseases [65]. In fact, ATXN3 has been shown to work with HTT, the mutated protein in Huntington’s disease, in a transcription-coupled repair (TCR) complex to repair DNA strand breaks [50,51,66]. However, the mutant form of ATXN3 binds and sequesters a protein of the TCR complex, preventing it from performing its role and leading to increased DNA strand breaks [50,53,66]. Evidence supporting this function has shown DNA damage to be increased in mouse models of SCA3, as measured by γ-H2AX signaling [50,61]. While we did not observe increased DNA damage via γ-H2AX expression in SCA3 oligodendrocytes at DIV5, we did observe γ-H2AX expression in *Atxn3*-KO cultures. Perhaps DNA damage is elicited by the loss-of-function of ATXN3 or the cellular stress of losing this protein. This does not preclude the possibility that oligodendrocytes in both SCA3 and *Atxn3*-KO oligodendrocytes may be prone to a different type of DNA damage. γ-H2AX is a marker of double-stranded DNA breaks [67], and because of high metabolic rates, high iron levels, and low glutathione levels, oligodendrocytes are particularly vulnerable to oxidative stress [48,49,68]. High oxidative stress can cause other types of DNA damage, including single-stranded DNA breaks and blocked DNA replication and transcription [69]. Future investigations of DNA damage in SCA3 oligodendrocytes should examine the relative contributions of various types of DNA damage to disease-associated oligodendrocyte signatures.

Of the canonical SCA3 disease-relevant pathways assessed in this study, none appeared to contribute to the cell-autonomous SCA3 oligodendrocyte maturation impairment. We therefore pivoted to evaluate the impact of mutant ATXN3 on characteristic pathways in oligodendrocyte differentiation. To understand what underlies the inability to mature in SCA3 oligodendrocytes, we probed for changes in the trimethylation of H3K9 and H3K27. This epigenetic regulatory modification is associated with the suppression of neuronal gene programs, enabling the continuation of oligodendrocyte differentiation [55]. The small magnitude of changes in H3K9me3 but not H3K27me3 was surprising but may point toward the need for more targeted investigations. Future studies should incorporate ChIP-seq to determine the methylation status of specific genes, since the immunocytochemistry results here represent global nuclear expression levels. Activation markers should also be investigated to determine whether the failed activation of oligodendrocyte maturation genes could be involved in the stalled differentiation process.

While the findings in this study are preliminary, they highlight the lack of understanding of common pathways in specific cell types within SCA3 disease. The use of immunocytochemistry alone is a major limitation to this study and future exploration of biochemical and histological mechanistic analysis will be necessary to investigate the function of mutant and normal ATXN3. For example, the assessment of the role ATXN3 plays in ubiquitin-proteasome pathways will be crucial, as our ubiquitinated proteins result in SCA3 and *Atxn3*-KO oligodendrocytes were opposing. Broader, unbiased approaches, such as proteomics or single-cell RNA sequencing, could be used to assess changes in OPC and oligodendrocyte protein or RNA expression to provide significant insight into the cell-specific pathomechanisms of SCA3 disease.

## 5. Conclusions

In this study, we established primary oligodendrocyte cell culture as a powerful mechanistc and translational model for many diseases with oligodendrocyte dysfunction, including SCA3, ALS, Alzheimer’s disease, and Huntington’s disease [10,21,22,23,24,25,26,27]. While many of the results presented here are contrary to what was expected, this also emphasizes the necessity of considering individual cell populations as isolated systems to study disease-relevant pathways. By culturing primary oligodendrocytes in a 2D system, advanced imaging and longitudinal cell tracking techniques can be utilized to further evaluate cell-specific disease-associated signatures. It is crucial to study the effects of disease on differentiation processes, protein quality control pathways, DNA damage signaling, and methylation modifications in isolated oligodendrocytes before we can fully comprehend how these mechanisms are influenced by extrinsic factors and contribute to disease in vivo. Understanding these interactions will offer insight into the cell-specific mechanisms driving disease pathogenesis.

## Figures and Tables

**Table 1:** List of primary antibodies used for oligodendrocyte immunocytochemistry.

Antibody	Host	Dilution	Company	Catalog #
ATXN3 (1H9)	Mouse IgG1	1:500	Millipore	MAB5360
SMOC1	Rabbit	1:100	ThermoFisher	PA5-31392
MBP	Rat	1:1000	Abcam	Ab7349
SQSTM1/p62	Mouse IgG2a	1:200	Abnova	89-015-843
BECLIN1	Rabbit	1:100	Abcam	Ab207612
SOX10	Mouse IgG1	1:100	Santa Cruz Biotechnology	sc-365692
SOX10	Rabbit	1:500	Cell Signaling	893565
Ubiquitinated Proteins	Mouse IgM	1:500	Millipore Sigma	04-262
Ki-67	Rat	1:300	ThermoFisher	14-5698-82
H3K27me3	Rabbit	1:500	Millipore	ABE44
H3K9me3	Rabbit	1:500	Abcam	ab8898
γ-H2AX	Mouse IgG1	1:500	Millipore	05-636

## Data Availability

The data reported here are available upon reasonable request.

## Supplementary Materials

The following are available online at https://www.mdpi.com/article/10.3390/cells11162615/s1, Figure S1: ATXN3 loss-of-function does not impact oligodendrocyte maturation. Figure S2: Protein ubiquitination is increased in *Atxn3*-KO OPCs, but not DIV5 oligodendrocytes. Figure S3: Loss of ATXN3 leads to upregulation of autophagy. Figure S4: DNA damage is increased in *Atxn3*-KO OPCs and immature oligodendrocytes, but not mature oligodendrocytes. Figure S5: Histone 3 methylation in oligodendrocytes is not affected by loss of ATXN3.

## References

[1] Seidel K., Siswanto S., Brunt E.R.P., Dunnen W.D., Korf H., Rueb U. (2012). Brain pathology of spinocerebellar ataxias. Acta Neuropathol..

[2] Rüb U., Schöls L., Paulson H., Auburger G., Kermer P., Jen J.C., Seidel K., Korf H., Deller T. (2013). Clinical features, neurogenetics and neuropathology of the polyglutamine spinocerebellar ataxias type 1, 2, 3, 6 and 7. Prog. Neurobiol..

[3] Paulson H.L., Shakkottai V.G., Clark H.B., Orr H. (2017). Polyglutamine spinocerebellar ataxias—From genes to potential treatments. Nat. Rev. Neurosci..

[4] Paulson H.L. (2007). Dominantly inherited ataxias: Lessons learned from Machado-Joseph disease/spinocerebellar ataxia type 3. Skull Base.

[5] Maciel P., Gaspar C., DeStefano A.L., Silveira I., Coutinho P., Radvany J., Dawson D.M., Sudarsky L., Guimarães J., E Loureiro J. (1995). Correlation between CAG repeat length and clinical features in Machado-Joseph disease. Am. J. Hum. Genet..

[6] Maruyama H., Nakamura S., Matsuyama Z., Sakai T., Doyu M., Sobue G., Seto M., Tsujihata M., Oh-I T., Nishio T. (1995). Molecular features of the CAG repeats and clinical manifestation of Machado--Joseph disease. Hum. Mol. Genet..

[7] Durr A., Stevanin G., Ms G.C., Duyckaerts C., Abbas N., Bs O.D., Chneiweiss H., Benomar A., Lyon-Caen O., Julien J. (1996). Spinocerebellar ataxia 3 and machado-joseph disease: Clinical, molecular, and neuropathological features. Ann. Neurol..

[8] Durr A. (2010). Autosomal dominant cerebellar ataxias: Polyglutamine expansions and beyond. Lancet Neurol..

[9] Gardiner S.L., Boogaard M.W., Trompet S., De Mutsert R., Rosendaal F.R., Gussekloo J., Jukema J.W., Roos R.A.C., Aziz N.A. (2019). Prevalence of carriers of intermediate and pathological polyglutamine disease–Associated alleles among large population-based cohorts. JAMA Neurol..

[10] McLoughlin H.S., Moore L.R., Paulson H.L. (2019). Pathogenesis of SCA3 and implications for other polyglutamine diseases. Neurobiol. Dis..

[11] Ichikawa Y., Goto J., Hattori M., Toyoda A., Ishii K., Jeong S.-Y., Hashida H., Masuda N., Ogata K., Kasai F. (2001). The genomic structure and expression of MJD, the Machado-Joseph disease gene. J. Hum. Genet..

[12] Zhang Y., Chen K., Sloan S.A., Bennett M.L., Scholze A.R., O’Keeffe S., Phatnani H.P., Guarnieri P., Caneda C., Ruderisch N. (2014). An RNA-sequencing transcriptome and splicing database of glia, neurons, and vascular cells of the cerebral cortex. J. Neurosci..

[13] Costa Mdo C., Paulson H.L. (2012). Toward understanding Machado-Joseph disease. Prog. Neurobiol..

[14] Paulson H., Perez M., Trottier Y., Trojanowski J., Subramony S., Das S., Vig P., Mandel J.-L., Fischbeck K., Pittman R. (1997). Intranuclear inclusions of expanded polyglutamine protein in spinocerebellar ataxia type 3. Neuron.

[15] Rüb U., Brunt E.R., Deller T. (2008). New insights into the pathoanatomy of spinocerebellar ataxia type 3 (Machado–Joseph disease). Curr. Opin. Neurol..

[16] Lukas C., Schöls L., Bellenberg B., Rüb U., Przuntek H., Schmid G., Köster O., Suchan B. (2006). Dissociation of grey and white matter reduction in spinocerebellar ataxia type 3 and 6: A voxel-based morphometry study. Neurosci. Lett..

[17] D’Abreu A., Jr M.F., Appenzeller S., Lopes-Cendes I., Cendes F. (2009). Axonal dysfunction in the deep white matter in Machado-Joseph disease. J. Neuroimaging.

[18] Guimaraes R.P., D’Abreu A., Yasuda C.L., Franca M.C., Silva B.H., Cappabianco F.A., Bergo F.P., Lopes-Cendes I.T., Cendes F. (2013). A multimodal evaluation of microstructural white matter damage in spinocerebellar ataxia type 3. Mov. Disord..

[19] Kang J.-S., Klein J.C., Baudrexel S., Deichmann R., Nolte D., Hilker R. (2013). White matter damage is related to ataxia severity in SCA3. J. Neurol..

[20] Rezende T.J.R., De Paiva J.L.R., Martinez A.R.M., Lopes-Cendes I., Pedroso J.L., Barsottini O.G.P., Cendes F., França M.C. (2018). Structural signature of SCA3: From presymptomatic to late disease stages. Ann. Neurol..

[21] Schuster K.H., Zalon A.J., Zhang H., DiFranco D.M., Stec N.R., Haque Z., Blumenstein K.G., Pierce A.M., Guan Y., Paulson H.L. (2022). Impaired oligodendrocyte maturation is an early feature in SCA3 disease pathogenesis. J. Neurosci..

[22] Philips T., Bento-Abreu A., Nonneman A., Haeck W., Staats K., Geelen V., Hersmus N., Küsters B., Van Den Bosch L., Van Damme P. (2013). Oligodendrocyte dysfunction in the pathogenesis of amyotrophic lateral sclerosis. Brain.

[23] Zhang P., Kishimoto Y., Grammatikakis I., Gottimukkala K., Cutler R.G., Zhang S., Abdelmohsen K., Bohr V.A., Misra Sen J., Gorospe M. (2019). Senolytic therapy alleviates Abeta-associated oligodendrocyte progenitor cell senescence and cognitive deficits in an Alzheimer’s disease model. Nat. Neurosci..

[24] Errea O., Rodriguez-Oroz M.C. (2021). Oligodendrocytes, a new player in the etiology of Parkinson’s disease. Mov. Disord..

[25] Ettle B., Schlachetzki J.C.M., Winkler J. (2016). Oligodendroglia and myelin in neurodegenerative diseases: More than just bystanders?. Mol. Neurobiol..

[26] Bardile C.F., Garcia-Miralles M., Caron N.S., Rayan N.A., Langley S.R., Harmston N., Rondelli A.M., Teo R.T.Y., Waltl S., Anderson L.M. (2019). Intrinsic mutant HTT-mediated defects in oligodendroglia cause myelination deficits and behavioral abnormalities in Huntington disease. Proc. Natl. Acad. Sci. USA.

[27] Kenigsbuch M., Bost P., Halevi S., Chang Y., Chen S., Ma Q., Hajbi R., Schwikowski B., Bodenmiller B., Fu H. (2022). A shared disease-associated oligodendrocyte signature among multiple CNS pathologies. Nat. Neurosci..

[28] Kang S.H., Li Y., Fukaya M., Lorenzini I., Cleveland D., Ostrow L., Rothstein J.D., E Bergles D. (2013). Degeneration and impaired regeneration of gray matter oligodendrocytes in amyotrophic lateral sclerosis. Nat. Neurosci..

[29] Huang B., Wei W., Wang G., Gaertig M.A., Feng Y., Wang W., Li X.-J., Li S. (2015). Mutant huntingtin downregulates myelin regulatory factor-mediated myelin gene expression and affects mature oligodendrocytes. Neuron.

[30] Osipovitch M., Martinez A.A., Mariani J.N., Cornwell A., Dhaliwal S., Zou L., Chandler-Militello D., Wang S., Li X., Benraiss S.-J. (2018). Human ESC-derived chimeric mouse models of Huntington’s disease reveal cell-intrinsic defects in glial progenitor cell differentiation. Cell Stem Cell.

[31] Benraiss A., Wang S., Herrlinger S., Li X., Chandler-Militello D., Mauceri J., Burm H.B., Toner M., Osipovitch M., Xu Q.J. (2016). Human glia can both induce and rescue aspects of disease phenotype in Huntington disease. Nat. Commun..

[32] Moore L.R., Rajpal G., Dillingham I.T., Qutob M., Blumenstein K.G., Gattis D., Hung G., Kordasiewicz H.B., Paulson H.L., McLoughlin H.S. (2017). Evaluation of antisense oligonucleotides targeting ATXN3 in SCA3 mouse models. Mol. Ther. Nucleic Acids.

[33] McLoughlin H.S., Moore L.R., Chopra R., Komlo R., McKenzie M., Blumenstein K.G., Zhao H., Kordasiewicz H.B., Shakkottai V.G., Paulson H.L. (2018). Oligonucleotide therapy mitigates disease in spinocerebellar ataxia type 3 mice. Ann. Neurol..

[34] Reina C.P., Nabet B.Y., Young P.D., Pittman R.N. (2012). Basal and stress-induced Hsp70 are modulated by ataxin-3. Cell Stress Chaperon.

[35] Jones T.R., Kang I.H., Wheeler D.B., A Lindquist R., Papallo A., Sabatini D.M., Golland P., E Carpenter A. (2008). CellProfiler Analyst: Data exploration and analysis software for complex image-based screens. BMC Bioinform..

[36] Schuster K.H., Zalon A.J., DiFranco D.M., Putka A.F., Stec N., Jarrah S., Naeem A., Haque Z., Zhang H., Guan Y. (2022). ASOs are an effective treatment for disease-associated oligodendrocyte signatures in premanifest and symptomatic SCA3 mice. bioRxiv.

[37] Dugas J.C., Tai Y.C., Speed T.P., Ngai J., Barres B.A. (2006). Functional genomic analysis of oligodendrocyte differentiation. J. Neurosci..

[38] Warrington A.E., Pfeiffer S.E. (1992). Proliferation and differentiation of O4+ oligodendrocytes in postnatal rat cerebellum: Analysis in unfixed tissue slices using anti-glycolipid antibodies. J. Neurosci. Res..

[39] Spitzer S.O., Sitnikov S., Kamen Y., Evans K.A., Kronenberg-Versteeg D., Dietmann S., de Faria O., Agathou S., Káradóttir R.T. (2019). Oligodendrocyte progenitor cells become regionally diverse and heterogeneous with age. Neuron.

[40] Hughes E.G., Stockton M.E. (2021). Premyelinating oligodendrocytes: Mechanisms underlying cell survival and integration. Front. Cell Dev. Biol..

[41] Richter-Landsberg C. (2008). The cytoskeleton in oligodendrocytes. Microtubule dynamics in health and disease. J. Mol. Neurosci..

[42] Barateiro A., Fernandes A. (2014). Temporal oligodendrocyte lineage progression: In vitro models of proliferation, differentiation and myelination. Biochim. Biophys. Acta.

[43] Stadelmann C., Timmler S., Barrantes-Freer A., Simons M. (2019). Myelin in the central nervous system: Structure, function, and pathology. Physiol. Rev..

[44] Liu W.J., Ye L., Huang W.F., Guo L.J., Xu Z.G., Wu H.L., Yang C., Liu H.F. (2016). p62 links the autophagy pathway and the ubiqutin–proteasome system upon ubiquitinated protein degradation. Cell. Mol. Biol. Lett..

[45] Ashkenazi A., Bento C.F., Ricketts T., Vicinanza M., Siddiqi F., Pavel M., Squitieri F., Hardenberg M., Imarisio S., Menzies F.M. (2017). Polyglutamine tracts regulate beclin 1-dependent autophagy. Nature.

[46] Nascimento-Ferreira I., Ferreira T., Sousa-Ferreira L., Auregan G., Onofre I., Alves S., Dufour N., Gould V.F.C., Koeppen A., Déglon N. (2011). Overexpression of the autophagic beclin-1 protein clears mutant ataxin-3 and alleviates Machado–Joseph disease. Brain.

[47] Herzog L.K., Kevei E., Marchante R., Böttcher C., Bindesbøll C., Lystad A.H., Pfeiffer A., Gierisch M.E., Salomons F.A., Simonsen A. (2019). The Machado–Joseph disease deubiquitylase ataxin-3 interacts with LC3C/GABARAP and promotes autophagy. Aging Cell.

[48] Tse K.-H., Herrup K. (2016). DNA damage in the oligodendrocyte lineage and its role in brain aging. Mech. Ageing Dev..

[49] French H.M., Reid M., Mamontov P., Simmons R.A., Grinspan J.B. (2009). Oxidative stress disrupts oligodendrocyte maturation. J. Neurosci. Res..

[50] Gao R., Liu Y., Silva-Fernandes A., Fang X., Paulucci-Holthauzen A., Chatterjee A., Zhang H.L., Matsuura T., Choudhary S., Ashizawa T. (2015). Inactivation of PNKP by mutant ATXN3 triggers apoptosis by activating the DNA damage-response pathway in SCA3. PLoS Genet..

[51] Gao R., Chakraborty A., Geater C., Pradhan S., Gordon K.L., Snowden J., Yuan S., Dickey A., Choudhary S., Ashizawa T. (2019). Mutant huntingtin impairs PNKP and ATXN3, disrupting DNA repair and transcription. eLife.

[52] Maiuri T., Suart C.E., Hung C.L.K., Graham K.J., Bazan C.A.B., Truant R. (2019). DNA damage repair in Huntington’s disease and other neurodegenerative diseases. Neurotherapeutics.

[53] Burma S., Chen B.P., Murphy M., Kurimasa A., Chen D.J. (2001). ATM Phosphorylates histone H2AX in response to DNA double-strand breaks. J. Biol. Chem..

[54] Sher F., Rößler R., Brouwer N., Balasubramaniyan V., Boddeke E., Copray S. (2008). Differentiation of neural stem cells into oligodendrocytes: Involvement of the polycomb group protein Ezh2. Stem Cells.

[55] Berry K., Wang J., Lu Q.R. (2020). Epigenetic regulation of oligodendrocyte myelination in developmental disorders and neurodegenerative diseases. F1000Research.

[56] Liu J., Magri L., Zhang F., Marsh N.O., Albrecht S., Huynh J.L., Kaur J., Kuhlmann T., Zhang W., Slesinger P.A. (2015). Chromatin landscape defined by repressive histone methylation during oligodendrocyte differentiation. J. Neurosci..

[57] Ramani B., Panwar B., Moore L.R., Wang B., Huang R., Guan Y., Paulson H.L. (2017). Comparison of spinocerebellar ataxia type 3 mouse models identifies early gain-of-function, cell-autonomous transcriptional changes in oligodendrocytes. Hum. Mol. Genet..

[58] Costa M.D.C., Radzwion M., McLoughlin H.S., Ashraf N.S., Fischer S., Shakkottai V.G., Maciel P., Paulson H.L., Öz G. (2020). In vivo molecular signatures of cerebellar pathology in spinocerebellar ataxia type 3. Mov. Disord..

[59] Haas E., Incebacak R.D., Hentrich T., Huridou C., Schmidt T., Casadei N., Maringer Y., Bahl C., Zimmermann F., Mills J.D. (2021). A Novel SCA3 knock-in mouse model mimics the human SCA3 disease phenotype including neuropathological, behavioral, and transcriptional abnormalities especially in oligodendrocytes. Mol. Neurobiol..

[60] Rodrigues A.-J., Costa M.D.C., Silva T.-L., Ferreira D., Bajanca F., Logarinho E., Maciel P. (2010). Absence of ataxin-3 leads to cytoskeletal disorganization and increased cell death. Biochim. Biophys. Acta.

[61] Wiatr K., Piasecki P., Marczak L., Wojciechowski P., Kurkowiak M., Ploski R., Rydzanicz M., Handschuh L., Jungverdorben J., Brustle O. (2019). Altered levels of proteins and phosphoproteins, in the absence of early causative transcriptional changes, shape the molecular pathogenesis in the brain of young presymptomatic Ki91 SCA3/MJD mouse. Mol. Neurobiol..

[62] Seiberlich V., Bauer N.G., Schwarz L., Ffrench-Constant C., Goldbaum O., Richter-Landsberg C. (2015). Downregulation of the microtubule associated protein Tau impairs process outgrowth and myelin basic protein mRNA transport in oligodendrocytes. Glia.

[63] Guo T., Noble W., Hanger D.P. (2017). Roles of tau protein in health and disease. Acta Neuropathol..

[64] Fu M.-M., McAlear T.S., Nguyen H., Oses-Prieto J.A., Valenzuela A., Shi R.D., Perrino J.J., Huang T.-T., Burlingame A.L., Bechstedt S. (2019). The Golgi outpost protein TPPP nucleates microtubules and is critical for myelination. Cell.

[65] Madabhushi R., Pan L., Tsai L.-H. (2014). DNA damage and its links to neurodegeneration. Neuron.

[66] Chatterjee A., Saha S., Chakraborty A., Silva-Fernandes A., Mandal S.M., Carvalho A., Liu Y., Pandita R.K., Hegde M., Hegde P.M. (2015). The role of the mammalian DNA end-processing enzyme polynucleotide kinase 3’-phosphatase in spinocerebellar ataxia type 3 pathogenesis. PLoS Genet..

[67] Kuo L.J., Yang L.-X. (2008). Gamma-H2AX—A novel biomarker for DNA double-strand breaks. In Vivo.

[68] Thorburne S.K., Juurlink B.H.J. (2002). Low glutathione and high iron govern the susceptibility of oligodendroglial precursors to oxidative stress. J. Neurochem..

[69] Jackson S.P., Bartek J. (2009). The DNA-damage response in human biology and disease. Nature.

