# Supplementary material for: Pathogenetic Mechanisms Underlying Spinocerebellar Ataxia Type 3 Are Altered in Primary Oligodendrocyte Culture
Source: Cells. 2022 Aug 22;11(16):2615. doi: 10.3390/cells11162615 (PMC9406561; doi:10.3390/cells11162615)

Supplemental Figure S1. ATXN3 loss-of-function does not impact oligodendrocyte maturation. Representative images of SMOC1 (green), MBP (red), and DAPI (blue) staining in *Atxn3*-KO oligodendrocytesat DIV0 (A) and DIV5 (B). Scale bar, 100um.

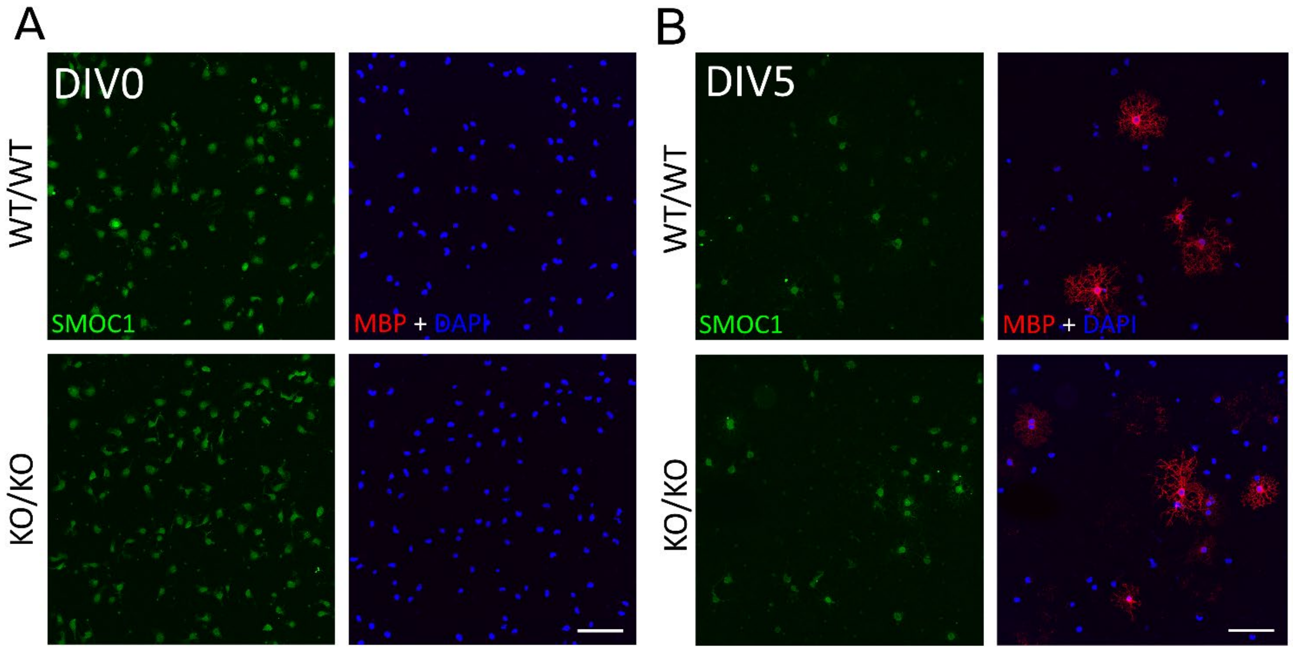

Supplemental Figure S2. Protein ubiquitination is increased in *Atxn3*-KO OPCs, but not mature oligodendrocytes. Representative images of Sox10 (cyan) and ubiquitinated (Ub) protein (red) staining in *Atxn3*-KO oligodendrocytes at DIV0 and DIV5. Scale bar: 100µm; inset scale bar: 12.5µm.

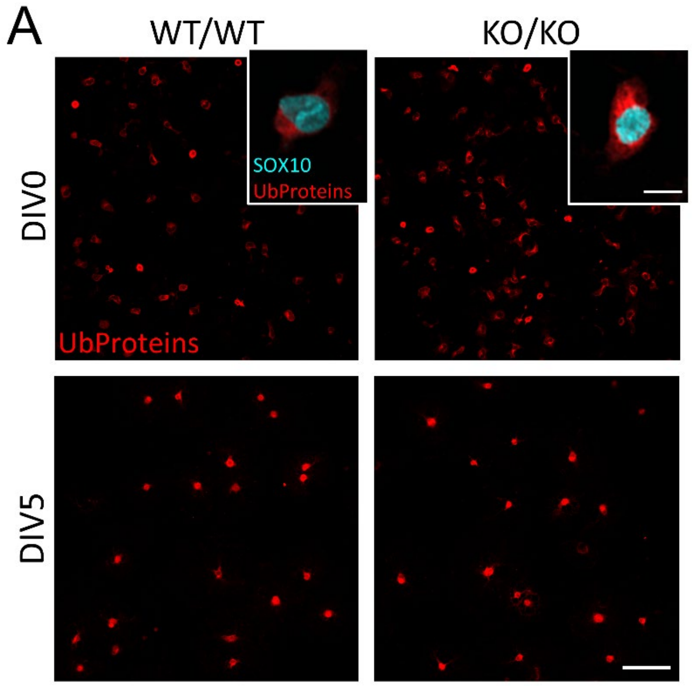

Supplemental Figure S3. Loss of ATXN3 leads to upregulation of autophagy. Representative images of p62 (A) and Beclin1 (B) staining in *Atxn3*-KO oligodendrocytes at DIV0 and DIV5. Scale bar: 100μm.

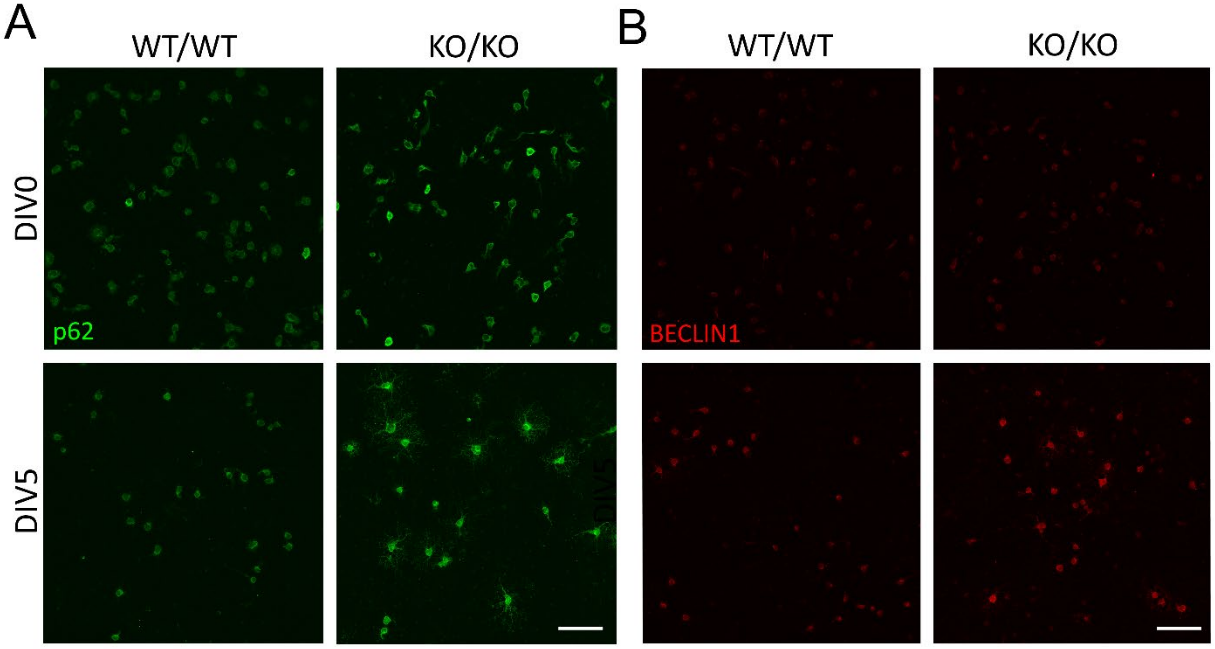

Supplemental Figure S4. DNA damage is increased in *Atxn3*-KO OPCs and immature oligodendrocytes, but not mature oligodendrocytes. Representative images of g-H2AX (white), Sox10 (cyan) and MBP (red) staining in *Atxn3*-KO oligodendrocytes at DIV0 (A) and DIV5 (B). Scale bar: 100µm; inset scale bar: 12.5µm.

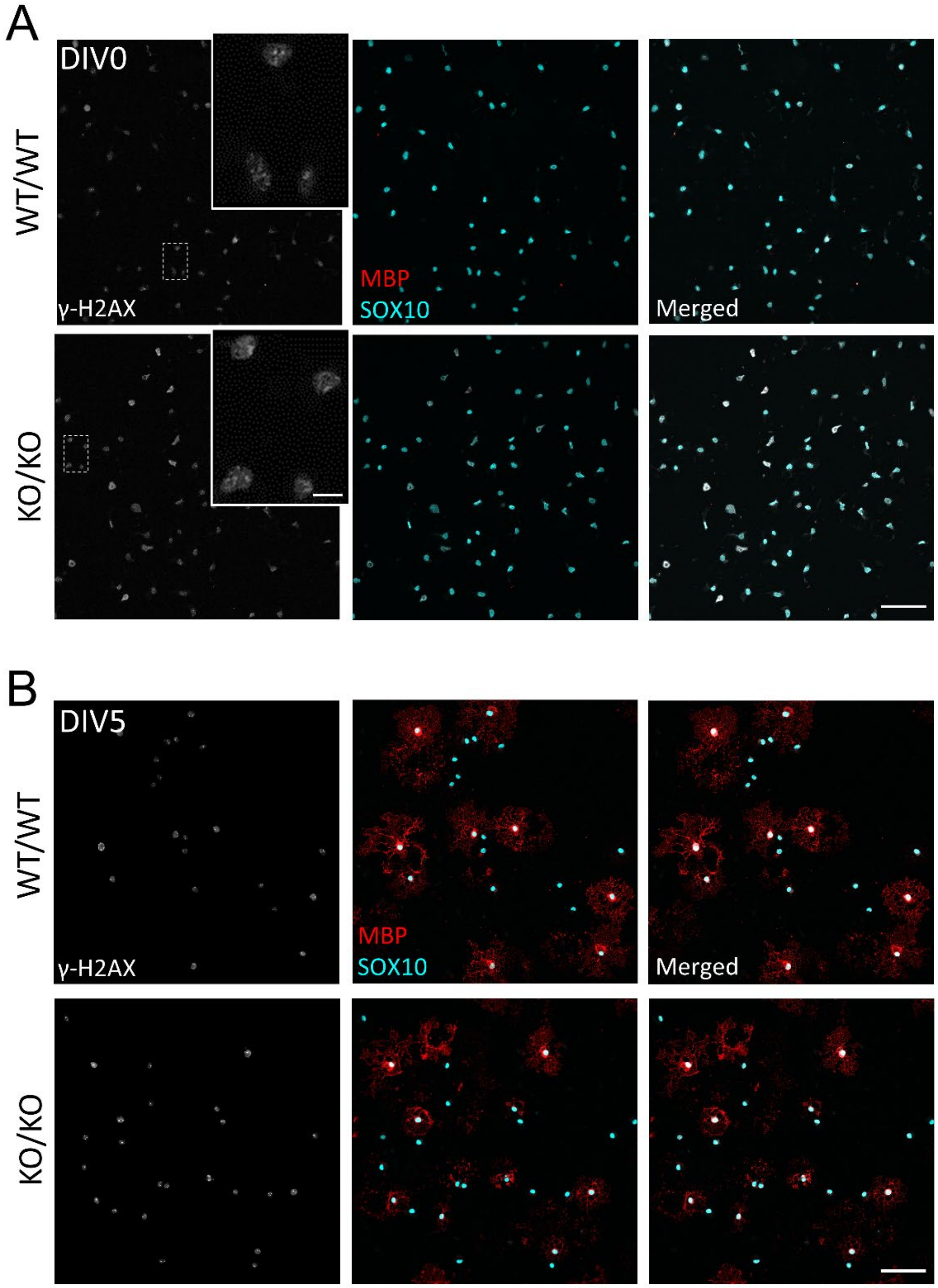

Supplemental Figure S5. Histone 3 methylation in oligodendrocytes is not affected by loss of ATXN3. (A-B) Representative images of H3K27me3 (magenta), Sox10 (cyan) and MBP (red) staining in *Atxn3*-KO oligodendrocytes at DIV0 (A) and DIV5 (B). (C-D) Representative images of H3K9me3 (magenta), Sox10 (cyan) and MBP (red) staining in *Atxn3*-KO oligodendrocytes at DIV0 (C) and DIV5 (D). Scale bar: 100μm.

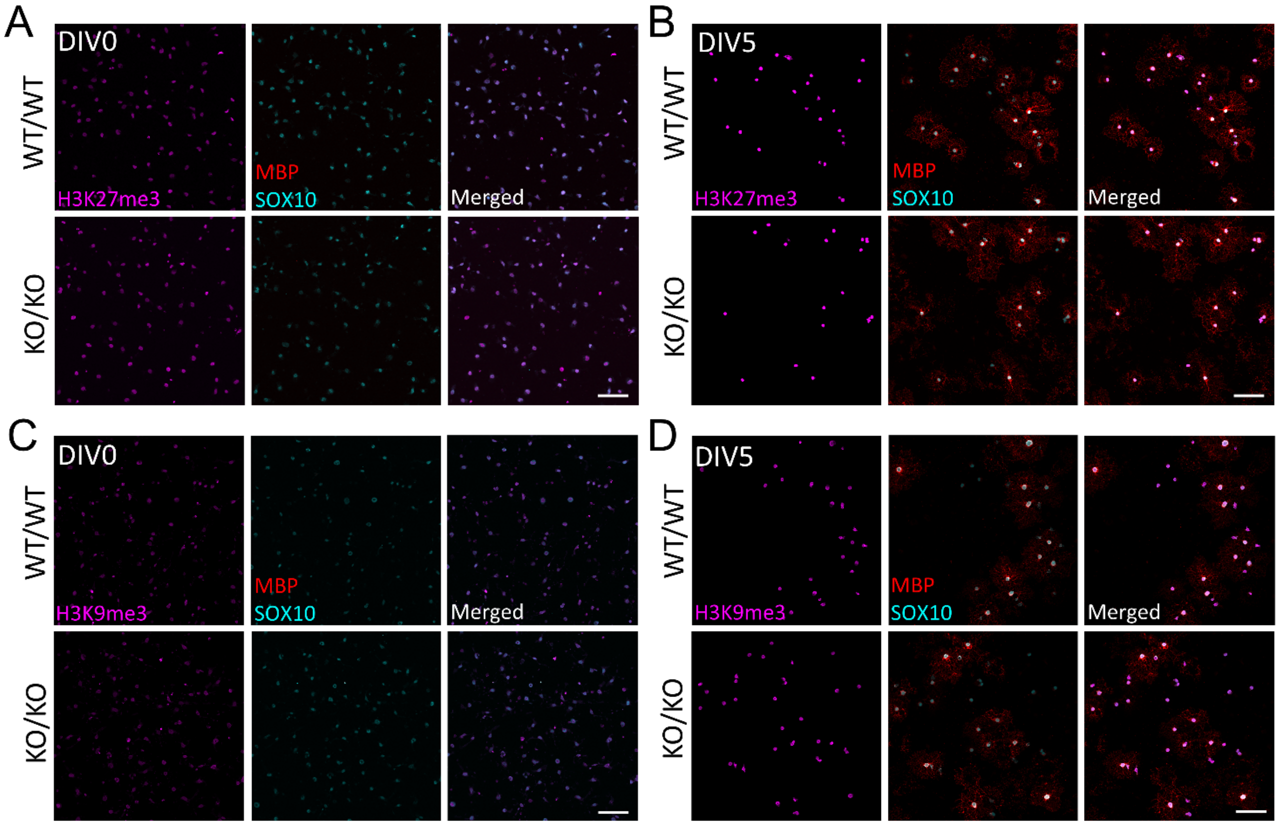

Supplement: Supplementary file 1 [file cells-11-02615-s001.zip › cells-1852688-supplementary.pdf]
